# Socio-economic inequalities in the use of flu vaccination in Europe: a multilevel approach

**DOI:** 10.1186/s13561-024-00535-1

**Published:** 2024-07-31

**Authors:** Dănuț-Vasile Jemna, Mihaela David, Liliane Bonnal, Cornel Oros

**Affiliations:** 1 Faculty of Economics and Business Administration, Alexandru Ioan Cuza University of Iaşi, Bld. Carol I, no. 22, 700506 Iași, Romania; 2 Laboratoire d’économie de Poitiers (LéP), University of Poitiers, Building A1, 2 Jean Carbonnier Street, TSA 81100, Poitiers Cedex 9, 86073 France

**Keywords:** Preventive healthcare, Influenza vaccination, Socio-economic inequalities, EHIS 2019, Multilevel logistic model.

## Abstract

**Background:**

The European-wide statistics show that the use of flu vaccination remains low and the differences between countries are significant, as are those between different population groups within each country. Considerable research has focused on explaining vaccination uptake in relation to socio-economic and demographic characteristics, health promotion and health behavior factors. Nevertheless, few studies have aimed to analyze between-country differences in the use of flu vaccination for the EU population. To address this gap, this study examines the socio-economic inequalities in the use of influenza vaccination for the population aged 15 years and over in all 27 EU Member States and two other non-EU countries (Iceland and Norway).

**Methods:**

Using data from the third wave of European Health Interview Survey (EHIS) 2019, we employed a multilevel logistic model with a random intercept for country, which allows controlling simultaneously the variations in individuals’ characteristics and macro-contextual factors which could influence the use of flu vaccination. In addition, the analysis considers the population stratified into four age groups, namely adolescents, young adults, adults and elderly, to better capture heterogeneities in flu vaccination uptake.

**Results:**

The main findings confirm the existence of socio-economic inequalities between individuals in different age groups, but also of significant variation between European countries, particularly for older people, in the use of influenza vaccination. In this respect, income and education are strong proxy of socio-economic status associated with flu vaccination uptake. Moreover, these disparities within each population group are also explained by area of residence and occupational status. Particularly for the elderly, the differences between individuals in vaccine utilization are also explained by country-level factors, such as the type of healthcare system adopted in each country, public funding, personal health expenditure burden, or the availability of generalist practitioners.

**Conclusions:**

Overall, our findings reveal that vaccination against seasonal influenza remains a critical public health intervention and bring attention to the relevance of conceiving and implementing context-specific strategies to ensure equitable access to vaccines for all EU citizens.

**Supplementary Information:**

The online version contains supplementary material available at 10.1186/s13561-024-00535-1.

## Background

The importance of prevention for building a healthy society and a well-functioning health system is underlined by numerous scientific studies and policies of public health organizations. Reducing inequalities in access to preventive healthcare is a priority for organizations such as WHO, as well as for EU [1] and national policies of European countries [2]. Despite this widespread view, the use of preventive health services is limited to a small percentage of the population [3]. Moreover, access to these services differs significantly among the European countries and, within countries, among different population groups. COVID-19 pandemic has indirectly contributed to disruptions in acute, primary and preventive healthcare to a variable extent [4]. On the other hand, a direct result of the public health measures and social restrictions imposed in response to COVID-19 has led to a huge drop-in influenza activity. Nevertheless, these measures are increasingly being mitigated [5]. Furthermore, several ongoing challenges related to the economic crisis, energy crisis and geo-political insecurity also have a negative impact on the population’s access to the use of preventive healthcare services.

Preventive healthcare services are important not only to avoid certain diseases, but also to identify existing health problems at an early stage, before they cause other issues or become more difficult to treat. This allows for more effective treatment in terms of having a greater impact on the health status of the population, but also in terms of saving total healthcare expenditure [6–8]. Despite the recognition of the cost-effectiveness of preventive measures, most health providers, including hospitals and physicians, do not prioritize preventive care services, but rather allocate their attention and a significant share of healthcare resources to disease management [3], thus spending much more on hospitals than on primary care [4]. For instance, before the pandemic, preventive healthcare in the EU accounted for 0.31% of GDP 2019, with the highest values in the UK and Italy, and the lowest percentages, below 0.1%, in Romania, Cyprus, and Slovakia. Relative to GDP, preventive healthcare expenditure in the EU increased to 0.38% in 2020 (ranging from 0.07%, recorded in Slovakia, to 0.52%, recorded in Italy and the Netherlands) and to 0.65% in 2021 (ranging from 0.12–1.25%, with Austria, the Netherlands and Denmark spending the highest amount on preventive healthcare, while Slovakia, Malta, and Poland recorded the lowest ratios). It is also worth mentioning that, at 6.0% (in 2021) and 3.5% (in 2020) of current healthcare expenditure across the EU, preventive healthcare was a notably greater function within the system of health than in 2019 when its share had been 2.9% of the total current healthcare expenditure. However, this increase occurs during the first and second calendar years of the COVID-19 crisis, reflecting the impact of the pandemic, particularly in the category ‘Immunisation programmes’ that includes vaccination campaign [9]. As a result, the share of immunisation programmes in total ‘preventive care’ expenditure increased from 13.7% in 2020 to 29.1% in 2021.

Even before the pandemic, leading governmental and expert organisations have consistently stressed the need to raise public awareness on the importance of prevention against influenza. The undoubted relevance of policy interventions at national and international level is supported by the fact that seasonal influenza affects all countries and causes 650,000 influenza-related deaths each year worldwide [5], and in Europe it continues to be a communicable disease with one of the highest impacts on population morbidity and mortality [10]. In this regard, the WHO recommends the following as the main strategies to reduce the morbidity and mortality associated with annual influenza: (a) strengthening disease surveillance and virological surveillance at both national and international levels; (b) increasing public awareness of the health and economic burden of influenza; (c) increasing the use of influenza vaccine; and (d) accelerating national and international action on pandemic preparedness [11]. Another important intervention is the 2009 Council Recommendation on seasonal influenza vaccination, which encourages EU Member States to adopt and implement action plans and policies to strengthen monitoring and surveillance systems at international, national and sub-national levels in order to ensure immunization of people who are more likely to develop serious illness if infected with influenza viruses [12]. In this regard, the Recommendation set an objective for EU Member States to achieve a 75% vaccination coverage rate with the seasonal influenza vaccine in key target groups, such as pregnant women at any stage of pregnancy, children aged 6 months to 5 years, older adults (over 65 years), people with chronic medical conditions, and healthcare workers. This objective is also targeted by the WHO, especially for the older people, this category being at greater risk of developing serious complications from influenza, including pneumonia and sepsis, which can result in serious illness or death [13]. However, in the EU, the proportion of the population aged 65 years and over that was vaccinated against influenza was just over half (50.8%) in 2021, with a range of 7.7% (Latvia) to 75.4% (Ireland). Between 2009 and 2021, the rate of vaccination against influenza in the EU varied, with the lowest rate recorded in 2015 at 40.0% and the highest recorded in 2009 at 54.6%. For other age groups,  during the 2018-2019 to 2020-2021 flu seasons, available data for a total of six European countries [12] show that the vaccination coverage rate was higher for older adults than for younger people, ranging from 9.2% (Luxembourg) to 25.1% (Iceland) among people aged 50–64. In the 18–49 age group, less than 10% of people were vaccinated in the reporting countries (Luxembourg, Norway and Iceland), while Slovakia had a vaccination coverage rate of around 3.8% among people aged 16–58. During the 2020–2021 season, an increasing trend has been observed in all age groups, but it is worth noting that this increase is supported to some extent by the impact of the COVID-19 pandemic [4].

Based on these Europe-wide statistics, uptake of influenza vaccination remains low, and the differences between countries are significant, as are those between different age groups within each country. Such evidence, together with the need to develop prevention policies at national and European level, underline the importance of research studies aiming to precisely identify the determinants explaining the differences between population groups in the use of flu vaccination.

### Related literature

There is an extensive literature examining the associations between the usage of medical-related preventive healthcare and individual and contextual factors. Socio-economic and demographic factors such as age, gender, education, income, marital status, area of residence are strong predictors of preventive care use [14–19]. Other important individual determinants of the use of preventive care include the presence of health problems, different types of chronic diseases or limitations in daily activities [2, 20, 21]. As argued by Jusot *et al.* [22], although the use of preventive services should be independent of an individual’s health status, some conditions may require more prevention, and in some circumstances these services may be part of a treatment. Additionally, a significant association is found between behavioral risk factors (smoking, physical inactivity, alcohol consumption, body weight) and the use of preventive health services [23–25]. Related to these particular health practices, Hoeck *et al.* [23] and Peytremann-Bridevaux and Santos-Eggimann [24] found that overweight and obesity among older adults were associated with higher odds of receiving influenza immunization, blood cholesterol measurement, or blood sugar measurement. At the same time, Peytremann-Bridevaux and Santos-Eggimann [24] point out that tobacco and alcohol consumption reduce the likelihood of visiting a generalist practitioner and dentist. Lastly, the lack of physical activity is associated with lower odds of using preventive services [25].

A general finding in the literature is that age is among the most important factors in the use of healthcare utilization [26] and the main indication for preventive reasons [2]. In this respect, empirical evidence shows that the likelihood of visiting a generalist [22], having cholesterol and blood sugar tests [27], getting a flu vaccination [22, 23, 28–31], attending cancer screenings [27, 32, 33], having a colonoscopy or stool blood test [22, 34], using regular dental checkups [14], or taking medical preventive healthcare [15] increases with age. In contrast, Borboudaki *et al.* [20] found that, as age increases, preventive use drops and healthcare access rises. In line with the authors’ results, age could also be considered a barrier to access to certain preventive services. For instance, Jusot *et al.* [22] show that all age groups have higher odds of visiting a specialist than those 80 years and over, as well as women aged 65–69 who have half the probability of having breast cancer screening than those aged 50. Another strand of literature focuses on socio-economic inequalities in the use of prevention services, usually driven by education and income levels [2, 7, 15, 20, 33, 35–37]. Despite the public health priority of ensuring equal access to prevention [38, 39], emerging evidence shows that income is an important contributor to unequal access to preventive healthcare. To this end, cross-country comparisons have revealed a general trend towards inequality in favor of the better-off, because a high income means a high payment capacity for healthcare. For instance, higher income groups show consistently higher levels of breast and colon cancer screening and influenza vaccination uptake in European countries [22, 27]. At the same time, there is strong evidence that education is closely related to the use of all types of dental services and preventive services in particular [40]. While Terraneo [41] found a clear pro-education gradient for visits to specialist physicians and dentists, he found no evidence of education disparities in the use of general practitioners. In line with previous research [42–45], the use of mammography, cervical cancer screening, or colorectal testing was more likely among women with higher educational attainment and higher household income. Carrieri and Wubker [2] also found education- and income-based disparities in blood tests and flu vaccination uptake. The authors underlined that people with lower incomes and who are less educated are more likely to seek preventive care late, for example when health shocks have occurred or when their health is deteriorating. Fewer studies underline that significant unequal utilization of preventive care services also exists among under-served immigrant and ethnic minority communities [33, 46] or between rural and urban areas [7], and not just by income and education.

Most studies analyzing socio-economic inequalities focus only on a particular age group, following the European recommendations on the use of different types of preventive services. In this respect, evidence of inequalities among the European countries has been found for the population aged 15 or 20 years and over [33, 47] or by age groups such as adolescents and young people [48], adults aged 25–64 [43–45, 49], adults aged 50–69 [22, 43, 45], people aged over 50 [2, 22, 24, 41, 42, 50–52], as well as the elderly [22, 51]. In the current context, these recommendations on prevention are being reviewed, including in terms of the age ranges that define the target groups, to strengthen prevention through early detection, e.g. screening for certain types of cancer [53] or flu vaccination [12]. As for the influenza virus, there are studies arguing that this global health threat causes serious illness among adolescents [54, 55], young adults [54–59], or older adults as well [55, 60] and it should not be neglected.

To explain these cross-national variations in the uptake of prevention, several scholars also propose macro-level indicators, such as public health expenditure, out-of-pocket expenditure, type of health system, number of doctors, number of hospital beds, or organised screening programmes [22, 33, 41, 61]. In addition, these factors also underscore the indirect barriers faced by socially disadvantaged groups [62]. Concerning the role of public funding, in countries where public health expenditure was higher, individuals had higher rates of generalist practitioners’ visits [22, 41], eye exams or colon cancer screening [22]. Analyzing the effect of hospital beds density, Terraneo [41] found a significant effect only on visits to specialists. The same author also identified that physicians’ density has a significant moderating effect on the association between education and the use of dental services. Jolidon *et al.* [43] show that in EU countries with higher expenditure in different social policy area (*i.e.* sickness/healthcare, disability, social exclusion, and public health) and a larger number of generalist practitioners, educational inequalities in cancer screening uptake - both Pap smear and mammography - were lower, while higher out-of-pocket payments had the opposite effect of increasing inequalities. According to Jusot *et al.* [22], no significant association was found between the share of out-of-pocket payments in total health expenditure and the use of any of the preventive services analyzed, including breast cancer screening. In another study [33], although almost all EU countries included have universal health coverage that covers direct costs for medical examinations – which describes in fact the Beveridge healthcare system, the authors point out that universal health coverage does not eliminate inequality in the use of screening for cancer and cardiovascular disease. Other features of healthcare systems such as general practitioner gatekeeping and stronger primary care systems were associated with reduced breast screening uptake [43]. In this respect, they can act as regulatory mechanisms, controlling and limiting specialist visits and possibly limiting unnecessary screening [63]. Regarding breast cancer screening, Wubker [61] also highlights other institutional factors that explain to a large extent the differences in screening rates between countries. His results indicate that the availability of a screening program, for example, increases the perceived benefits of screening, and therefore the reduction in mortality over time. As has been shown in other studies [44, 64], socio-economic disparities in cervical cancer screening participation were significantly lower in countries with high accessibility to healthcare and even lower if these countries also had an organized screening program. Willems *et al.* [45] also found that the educational gradient in cancer screening participation prevails in contexts of higher macro-level gender inequality, regardless of whether or not countries have an organized screening program.

### Aim and contribution

In the case of influenza immunization, considerable research has focused on explaining vaccination uptake in relation to socio-economic and demographic characteristics, health promotion and health behavior factors. Nevertheless, few studies have aimed to analyze between-country differences in the use of flu vaccination for the EU population. To address this gap, the current study examines the socio-economic inequalities in the use of influenza vaccination for the population aged 15 years and over in all 27 EU Member States and two other non-EU countries (Iceland and Norway). These inequalities are assessed by differences between individual socio-economic characteristics of the population and country-level institutional characteristics using data from the European Health Interview Survey (EHIS) 2019.

This study makes several key contributions to the existing literature. First, our paper represents, to the best of our knowledge, the first study using data from the third wave of EHIS to explore the inequalities in the use of flu vaccination in EU. The EHIS provides comparable data on health status, healthcare utilization, health determinants and background socio-economic variables of the population aged 15 years and over in all EU countries. Second, our paper investigates the variations in flu vaccination use among the entire population covered in the survey, whereas the existing literature focuses mainly on people aged 65 years and older, being one of the most at risk groups. However, international public health organizations also argue that it is important for the entire population to get vaccinated against influenza every year for at least two reasons: first, immunity (protection) decreases over time; second, flu viruses are constantly changing, so the vaccine is updated frequently to provide the best protection. Therefore, our study takes into account four age groups (adolescents, young adults, older adults, and elderly) to provide an in-depth analysis of heterogeneities in flu vaccination uptake in EU countries. Finally, our study, by evaluating differences in inequality both between age groups and between EU countries, can contribute to emphasizing this issue on the policy agenda for health promotion, early disease prevention and self-care practices as an integrated part of health system responses and people’s everyday lives starting from a young age.

## Methods

### Data

The data used in this study are from the third wave of EHIS, conducted in 2019. The reference population is represented by persons aged 15 years and over, living in private households in each EU Member State at the time of data collection [65]. The standardized EHIS questionnaire, translated into the national languages of the 27 Member States, was used either by self-administration, face-to-face interviews or telephone interviews. Microdata was collected using nationally representative probability samples [66]. Although the survey was conducted for all 28 EU members at 2019 level, the anonymized data provided by Eurostat does not contain the sample for the UK. In addition, in the third wave of EHIS data was collected for 4 more countries, of which Iceland and Norway are available for our analysis. After omitting all cases with missing information, *i.e.* 60,057 (18.45%), the final sample consisted of 265,520 survey participants.

The macro-level data used to analyze cross-country differences are gathered from Eurostat. Descriptive statistics of all individual and country-level characteristics for the 29 countries included in this study are provided in Tables A1a-b, A2, and A3 (Appendix).

### Selection of variables

The classic structure of the EHIS questionnaire has four sections with four categories of variables: social core, health, healthcare utilization, health determinants. The variables of interest for our analysis are briefly presented below.

#### Dependent variable

According to EU recommendations [67], the flu vaccine should be re-administered annually to ensure it remains effective, as seasonal flu viruses evolve each year. In the EHIS, data about vaccination are self-reported. The individuals were asked to answer the question: “When was the last time you’ve been vaccinated against flu?”. The intention of the question is to find out how many people are protected against seasonal influenza [66], but it is not possible to distinguish between individuals who have been vaccinated on their own initiative and those who have followed their doctor’s recommendation. Asking for the month and the year of the last vaccination and recording the date on which each respondent was interviewed enabled to construct the indicator on influenza vaccination in the last 12 months or during the last season. Furthermore, the response categories “Too long ago (before last year)” and “Never” have been merged and also represent the category of respondents who do not comply with the recommendation for influenza vaccination in the last 12 months. Therefore, we recoded these answer categories into a binary variable: those who have received influenza vaccine during the last 12 months (coded as 1) against those who did not have the vaccine during the reference period (coded as 0).

#### Individual independent variables

According to the literature [7, 41, 68], we group the individuals’ characteristics into four categories, namely predisposing factors, enabling factors, health status, and health behavior.

- *Predisposing factors* are age, gender, marital status and area of residence. For the entire population, age is considered in ten-year intervals. Models are also constructed for four different age groups. For adolescents, the age range 15–19 years was considered. Adults were grouped into two age groups: young adults (20–44) and adults (45–64). Finally, with respect to elderly, the range 65 years and over was considered. The marital status was sorted into four categories: never married, married, widowed, divorced. The area of residence can be a city, town and suburb, or rural area.
- *Enabling factors* include education, household income, and employment status. The education level was measured based on the last degree obtained by respondents and according to the ISCED 2011 classification into three categories: primary, secondary and tertiary. Income is defined by quantiles. Employment status is expressed as a variable with four categories, indicating if respondents are employed, unemployed, retired, or if they are in another situation (including unable to work due to long term health problems; student or pupil; performing domestic duties; compulsory military or civilian service; other).
- *Health Status* includes self-perceived general health and health-related conditions such as limitations, chronic conditions, and depression. For self-perceived health status, respondents were grouped in four categories: bad, fair, good, and very good. In the case of limitations in activities because of health problems, individuals were grouped into three categories: severely limited, limited but not severely limited, and not limited at all. The variables controlling the existence of specific diseases and chronic conditions are binary (yes and no). In this respect, the health status of individuals is considered according to the age group of individuals. Therefore, for adolescents, we controlled only the self-perceived general health, whereas for the other age groups, we considered other health conditions such as: limitations in daily activities, asthma, bronchitis, blood pressure, coronary disease, heart attack, stroke, arthrosis, diabetes, bladder and kidney problems, and also depression. It should be mentioned that the latter variables were used in different combinations from one age group to another and that self-perceived general health was excluded due to possible association with the other health-related conditions. On the one hand, these variables have been chosen considering the fact that influenza can worsen the symptoms of certain chronic diseases [13], and thus requiring more prevention, such as flu vaccination [22]. On the other hand, it is well known that there is an important link between ageing and many chronic conditions, with ageing increasing the risk of many common diseases such as diabetes, cardiovascular disease, chronic obstructive pulmonary disease, neurodegenerative diseases, osteoporosis, *etc.* [69].
- *Health Behavior* encompasses the factors Body Mass Index (BMI), smoking, physical activity and diet. Based on BMI, the population was divided into four categories: underweight, normal weight, overweight, and obese. For smoking behavior four categories are defined: daily smoker, occasional smoker, former daily smoker, and non-smoker (*i.e.* those who have never smoked). Concerning physical activity level, individuals were categorized as inactive, low active, moderately active, or highly active, using the MET (metabolic equivalent) score (IPAQ-SF). With respect to respondents’ nutritional behavior consumption, the number of fruits and vegetables consumed by a person per day was considered. In this respect, for a healthy diet, the WHO [70] recommends consuming at least 400 g (*i.e.* five portions) of fruit and vegetables per day. Based on these guidelines and the data available in the survey, we constructed the variable on dietary habits that divides the respondents into three categories: sufficient (at least 5 fruits and/or vegetables once or more a day), moderate (less than 5 fruits and/or vegetables once or more a day or 4 to 6 times a week), insufficient (1 to 3 times a week or less than once a week or never). Regarding alcohol consumption, due to missing data for Italy, the variable was not included in this analysis.

#### Country-level variables

In line with previous research [22], the country-level confounders used in the present study are public health expenditure (calculated as the share of GDP), out-of-pocket expenditure as percent from total current health expenditure, number of generalist and specialist practitioners per 100,000 inhabitants, and the type of healthcare system. For health systems in the EU-27, two main models are adopted: Beveridge (Cyprus, Denmark, Spain, Finland, Ireland, Iceland, Italy, Latvia, Malta, Portugal, Sweden, and Norway) and Bismarck (Austria, Belgium, France, Germany, Greece, Luxembourg, Netherlands, Bulgaria, Croatia, Czech Republic, Hungary, Poland, Romania, Slovakia, Estonia, Lithuania, and Slovenia). The major difference between the two models lies in the way the health system is financed. In the Beveridge system, healthcare is provided and financed by the government through tax payments. The Bismarck system uses an insurance system, usually financed jointly by employers and employees through payroll deduction. Further, we consider other relevant features describing the healthcare systems in the 27 countries of the EU [71], which refer to the portfolio of services, *i.e.* if it is defined at central level or not, to the primary care, *i.e.* if the patient must be registered with a doctor or not, and to the existence of some copayment by users in primary care.

### Statistical analyses

Pooling data across the EU countries, we employed a multilevel logistic model with a random intercept for country, which allows controlling simultaneously the variations in individuals’ characteristics and macro-contextual factors that could influence the use of flu vaccination [72].

The random-intercept model for a binary response can be expressed by the following regression equation [73]:

$$\:{y}_{ij}={p}_{ij}+{\epsilon\:}_{ij}$$

$$\:logit\left({p}_{ij}\right)={\beta\:}_{0j}+{X}_{ij}\beta\:+{Z}_{j}\gamma\:$$

$$\:{{\beta\:}_{0j}={\beta\:}_{0}+u}_{j}$$

For dependent variable the values $\:{y}_{ij}$ for any individual *i* from a country *j* take 1 if the individual uses a preventive service and 0 otherwise, and $\:{p}_{ij}=P({y}_{ij}=1)$. $\:{X}_{ij}$ are the independent variable at individual level, and $\:{Z}_{j}$ are specific country-level variables. $\:{\beta\:}_{0j}$ represents the random intercepts for countries, where $\:{\beta\:}_{0}$ is the mean of the country intercepts that capture the differences between countries in the average level of flu vaccination use. The random errors are $\:{u}_{j}$ and capture the unobserved country-specific factors that cause differences in flu vaccination uptake across countries [22]. It is assumed that these are normal distributed with zero mean and $\:{\sigma\:}_{u}^{2}$ variance. The estimate for $\:{\sigma\:}_{u}^{2}$ represents a measure for the differences across the countries in the preventive healthcare utilization, after controlling for independent variables. The individual-level random error $\:{\epsilon\:}_{ij}$ captures unobserved individual factors that might explain individual differences in flu vaccination use within a country. In the equivalent threshold model, $\:{\epsilon\:}_{ij}$ have a standard logistic distribution with zero mean and $\:{\sigma\:}_{\epsilon\:}^{2}$ variance, which is $\:{\pi\:}^{2}/3$ or approximately 3.29 [74].

Using this methodology, the empirical strategy of the study consists of several steps. Thus, considering that age is an important predictor of most of the healthcare services, the analysis is conducted for each age group: adolescent (15–19 years), young adults (20–44), adults (45–64), and elderly (65 years and over). Moreover, in order to better capture the effect of socio-economic inequalities between individuals according to different age groups, several regression models are employed following the stepwise procedure [18], which involves considering in the first step only variables related to the individual’s socio-economic status (age, gender, education, income, professional status, area of residence), adding, in the second step, health behavior factors (BMI, smoking, diet, physical activity), and including, in the third step, health status factors (different chronic conditions or health problems according to each age group). For each model, the variance partition coefficient (VPC) was calculated to assess between-country differences in vaccine uptake [73, 75]. Further, separate models were built for each country-level variable - because of the low degrees of freedom at country level [22] - and adjusted for all individuals’ characteristics to assess the association between macro-contextual factors and influenza vaccine uptake. In line with Jolidon* et al.* [43], for the latter models the VPC was computed to estimate the relative importance of macro-level factors, *i.e.* to assess how each of these variables contributes to explaining the higher-level variance of the model.

It should also be mentioned that the variance inflation factors (VIF) were below 3 for all variables, indicating that there were no multicollinearity issues.

## Results

### Association between the use of flu vaccination and individual factors

Tables 1, 2, 3 and 4 present the individual determinants of the probability of flu vaccination in all 29 European countries considered, and separately for each age group. Estimated coefficients were translated into odds ratios (OR) for facilitating the interpretation.

#### Adolescents

Among the adolescents’ group (15–19 years), the results indicate few factors that significantly explain the probability of getting a flu vaccine (Table 1).

In this respect, individuals with the highest level of income, residing in cities, and having a very good health condition were more likely to use influenza vaccination in the past 12 months. In contrast, other socio-demographic factors such as gender and education, as well as determinants of health behavior, are not significantly associated with the probability that an adolescent will get vaccinated against influenza.

**Table 1:** The association between flu vaccination and its determinants among adolescents

Variables	Model 1		Model 2		Model 3	
Fixed Effects	OR	*p*-value	OR	*p*-value	OR	*p*-value
Intercept	0.06	**< 0.001**	0.04	**< 0.001**	0.05	**< 0.001**
Sex (reference: *Male*)						
Female	0.94	0.458	0.92	0.279	0.90	0.188
Education (reference: *Primary*)						
Secondary lower	0.75	0.075	0.76	0.062	0.76	0.240
Secondary upper	0.79	0.082	0.81	0.126	0.82	0.150
Income (reference: *< Q1*)						
Q1-Q2	1.03	0.823	1.03	0.832	1.03	0.813
Q2-Q3	1.11	0.400	1.09	0.492	1.09	0.456
Q3-Q4	1.12	0.361	1.10	0.463	1.11	0.411
Q4-Q5	1.32	**0.032**	1.29	**0.052**	1.30	**0.047**
Area of residence (reference: *Rural areas*)						
Cities	1.33	**0.004**	1.33	**0.004**	1.33	**0.004**
Towns and suburbs	1.13	0.236	1.13	0.237	1.13	0.232
BMI (reference: *Normal weight*)						
Underweight			1.06	0.633	1.05	0.707
Overweight			0.96	0.701	0.93	0.483
Smoking (reference: *Daily*)						
Former			1.20	0.551	1.22	0.507
Occasional			0.97	0.977	0.98	0.928
Never			1.17	0.309	1.22	0.206
Diet (reference: *Insufficient*)						
Moderate			0.98	0.896	1.00	0.994
Sufficient			1.20	0.189	1.24	0.127
Physical activity (reference: *Inactive*)						
Low			1.36	0.214	1.36	0.219
Moderate			1.19	0.436	1.21	0.399
High			1.22	0.380	1.26	0.306
Self-perceived health (reference: * Fair*)						
Good					0.79	0.102
Very good					0.66	**0.005**
**Random Effects**						
VPC*	0.156		0.156		0.154	
N _COUNTRY_	29		29		29	
Observations	12,396		12,396		12,396	
Marginal R^2^ / Conditional R^2^	0.008 / 0.166		0.013 / 0.170		0.017 / 0.171	

*Notes*: *Variance partitioning coefficient

#### Young adults

The determinants on utilization of flu vaccines among young adults (20–44 years) are presented in Table 2.

**Table 2:** The association between flu vaccination and its determinants among young adults

Variables	Model 1		Model 2		Model 3	
Fixed Effects	OR	*p*-value	OR	*p*-value	OR	*p*-value
Intercept	0.03	**< 0.001**	0.02	**< 0.001**	0.11	**< 0.001**
Sex (reference: *Male*)						
Female	1.22	**< 0.001**	1.19	**< 0.001**	1.17	**< 0.001**
Age (reference: *20–24 years*)						
25–29 years	0.92	0.133	0.93	0.181	0.90	0.057
30–34 years	0.99	0.899	1.00	0.996	0.96	0.417
35–39 years	1.03	0.576	1.04	0.513	0.98	0.674
40–44 years	1.05	0.386	1.05	0.401	0.97	0.579
Education (reference: *Primary*)						
Secondary	1.01	0.880	0.99	0.948	1.02	0.824
Tertiary	1.27	**< 0.001**	1.41	**< 0.001**	1.47	**< 0.001**
Marital status (reference: *Married*)						
Divorced	0.85	**0.023**	0.88	0.080	0.86	**0.033**
Never	0.83	**< 0.001**	0.85	**< 0.001**	0.83	**< 0.001**
Employment (reference: *Unemployed*)						
Employed	1.27	**< 0.001**	1.26	**0.001**	1.30	**< 0.001**
Other	1.24	**0.003**	1.20	**0.013**	1.17	**0.034**
Income (reference: *< Q1*)						
Q1-Q2	1.07	0.207	1.06	0.250	1.07	0.174
Q2-Q3	1.15	**0.006**	1.14	**0.012**	1.16	**0.005**
Q3-Q4	1.28	**< 0.001**	1.26	**< 0.001**	1.28	**< 0.001**
Q4-Q5	1.55	**< 0.001**	1.52	**< 0.001**	1.55	**< 0.001**
Area of residence (reference: *Rural areas*)						
Cities	1.31	**< 0.001**	1.31	**< 0.001**	1.29	**< 0.001**
Towns and suburbs	1.12	**0.003**	1.12	**0.003**	1.12	**0.006**
BMI (reference: *Normal weight*)						
Underweight			1.04	0.612	1.03	0.721
Overweight			1.04	0.209	1.01	0.747
Obese			1.27	**< 0.001**	1.13	**0.005**
Smoking (reference: *Daily*)						
Former			1.25	**< 0.001**	1.25	**< 0.001**
Occasional			1.13	0.056	1.15	**0.030**
Never			1.31	**< 0.001**	1.33	**< 0.001**
Diet (reference: *Insufficient*)						
Moderate			1.11	0.088	1.12	0.058
Sufficient			1.28	**< 0.001**	1.30	**< 0.001**
Physical activity (reference: *Inactive*)						
Low			1.14	**0.042**	1.17	**0.019**
Moderate			1.12	**0.046**	1.15	**0.013**
High			1.21	**0.001**	1.26	**< 0.001**
Limitations (reference: *Not limited*)						
Limited					1.32	**< 0.001**
Severely limited					1.57	**< 0.001**
Asthma (reference: *Yes*)						
No					0.52	**< 0.001**
Blood pressure (reference: *Yes*)						
No					0.72	**< 0.001**
Diabetes (reference: *Yes*)						
No					0.40	**< 0.001**
Depression (reference: *Yes*)						
No					1.06	0.344
**Random Effects**						
VPC*	0.160		0.158		0.156	
N _COUNTRY_	29		29		29	
Observations	81,463		81,463		81,463	
Marginal R^2^ / Conditional R^2^	0.035 / 0.190		0.043 / 0.194		0.057 / 0.204	

*Notes*: *Variance partitioning coefficient

Across the EU countries, on average, women are more likely to get a flu vaccine. Individuals in different age groups display no difference in the probability of using influenza vaccination. The effect of other socio-economic determinants reveals that individuals with higher education, higher income, being married, having a job, and residing in cities or towns and suburbs had higher odds of getting flu vaccine in the past 12 months. Turning to the personal health practices of young adults, the findings indicate that smoking reduces the probability of using flu vaccination. Also, those who have problems with obesity, do not take care of their diet or do not practice any physical activity are less likely to get a flu vaccine. Need predictors have a strong association with flu vaccine use. In this respect, individuals moderately or severely limited in usual activities because of health problems are more likely to be preventive than those in good health. Moreover, those suffering from asthma, blood pressure or diabetes have higher probability to get influenza vaccination than individuals without these health problems. However, the association between depression and flu vaccination among younger adults is not statistically significant.

#### Adults

After adjusting for socio-economic determinants, followed by health behavior and health status factors related to flu vaccination utilization, the results of the regression analysis pertaining to adults’ age group (45–64 years) are shown in Table 3.

**Table 3:** The association between flu vaccination and its determinants among adults

Variables	Model 1		Model 2		Model 3	
Fixed Effects	OR	*p*-value	OR	*p*-value	OR	*p*-value
Intercept	0.07	**< 0.001**	0.04	**< 0.001**	0.29	**< 0.001**
Sex (reference: *Male*)						
Female	1.08	**< 0.001**	1.07	**0.001**	1.09	**< 0.001**
Age (reference: *45–49 years*)						
50–54 years	1.17	**< 0.001**	1.16	**< 0.001**	1.11	**0.002**
55–59 years	1.62	**< 0.001**	1.58	**< 0.001**	1.43	**< 0.001**
60–64 years	2.31	**< 0.001**	2.24	**< 0.001**	2.00	**< 0.001**
Education (reference: *Primary*)						
Secondary	0.86	**< 0.001**	0.86	**< 0.001**	0.90	**0.011**
Tertiary	1.14	**0.002**	1.13	**0.004**	1.23	**< 0.001**
Marital status (reference: *Married*)						
Divorced	0.88	**< 0.001**	0.92	**0.004**	0.87	**< 0.001**
Widowed	0.99	0.831	1.01	0.864	0.96	0.463
Never	1.04	0.216	1.07	**0.018**	1.04	0.147
Employment (reference: *Unemployed*)						
Employed	1.11	**0.027**	1.09	0.091	1.18	**0.001**
Retired	1.34	**< 0.001**	1.30	**< 0.001**	1.24	**< 0.001**
Other	1.54	**< 0.001**	1.50	**< 0.001**	1.31	**< 0.001**
Income (reference: *< Q1*)						
Q1-Q2	0.96	0.306	0.95	0.198	0.99	0.738
Q2-Q3	1.04	0.314	1.02	0.537	1.08	**0.031**
Q3-Q4	1.12	**0.002**	1.10	**0.008**	1.20	**< 0.001**
Q4-Q5	1.23	**< 0.001**	1.22	**< 0.001**	1.36	**< 0.001**
Area of residence (reference: *Rural areas*)						
Cities	1.19	**< 0.001**	1.21	**< 0.001**	1.17	**< 0.001**
Towns and suburbs	1.08	**0.003**	1.09	**0.001**	1.07	**0.014**
BMI (reference: *Normal weight*)						
Underweight			0.96	0.655	0.93	0.450
Overweight			1.11	**< 0.001**	1.03	0.206
Obese			1.37	**< 0.001**	1.06	**0.042**
Smoking (reference: *Daily*)						
Former			1.29	**< 0.001**	1.29	**< 0.001**
Occasional			1.03	0.557	1.07	0.268
Never			1.21	**< 0.001**	1.27	**< 0.001**
Diet (reference: *Insufficient*)						
Moderate			1.13	**0.007**	1.16	**0.002**
Sufficient			1.32	**< 0.001**	1.36	**< 0.001**
Physical activity (reference: *Inactive*)						
Low			0.97	0.410	1.03	0.446
Moderate			0.98	0.629	1.09	**0.009**
High			0.94	0.117	1.10	**0.016**
Limitations (reference: *Not limited*)						
Limited					1.42	**< 0.001**
Severely limited					1.66	**< 0.001**
Asthma (reference: *Yes*)						
No					0.57	**< 0.001**
Bronchitis (reference: *Yes*)						
No					0.59	**< 0.001**
Blood pressure (reference: *Yes*)						
No					0.76	**< 0.001**
Coronary (reference: *Yes*)						
No					0.72	**< 0.001**
Diabetes (reference: *Yes*)						
No					0.52	**< 0.001**
Depression (reference: *Yes*)						
No					0.94	0.116
**Random Effects**						
VPC*	0.148		0.147		0.147	
N _COUNTRY_	29		29		29	
Observations	93,720		93,720		93,720	
Marginal R^2^ / Conditional R^2^	0.043 / 0.173		0.052 / 0.179		0.084 / 0.220	

*Notes*: *Variance partitioning coefficient

Similar to young adults, women have a higher probability of flu vaccine uptake. In addition, as expected, the likelihood of being vaccinated against influenza increases with age, and this increase is even more evident in the adults group. Individuals with a higher level of education have a higher probability of getting the flu vaccine, compared to their counterparts with lower education. Surprisingly, individuals with secondary level of education are less likely to get the vaccine than those with primary education. Considering marital status, the results show that divorced adults are less likely to be vaccinated against flu than married adults, but between the latter and widowed or unmarried adults there are no significant differences. Adults in different employment and income groups display significant differences in the probability of using flu vaccination, which is higher in comparison to the unemployed and those with the lowest income, respectively. The area of residence is another significant determinant showing that individuals from rural area are the least preventive when it comes to using the flu vaccine. Considering health behavioral factors, adults with weight problems, who smoke daily, have an unbalanced diet or are physically inactive are least likely to use the flu vaccine. Moreover, those having difficulties in activities of daily living and having chronic health conditions such as asthma, bronchitis, blood pressure, coronary artery disease, and diabetes have a higher probability of getting a flu vaccination. However, as in the case of young adults, whether or not being depressed is not associated with an adult’s likelihood of getting the flu vaccine.

#### Elderly

With respect to the elderly age group (65 years and more), the significant determinants of flu vaccination use at the individual level are in general similar to those obtained for young and older adults. However, the direction and especially the magnitude of the association between the probability of getting a flu vaccination and some of these factors differs compared with the other age groups (Table 4).

**Table 4:** The association between flu vaccination and its determinants among elderly

Variables	Model 1		Model 2		Model 3	
Fixed Effects	OR	*p*-value	OR	*p*-value	OR	*p*-value
Intercept	0.28	**< 0.001**	0.15	**< 0.001**	0.72	0.133
Sex (reference: *Male*)						
Female	0.98	0.336	1.00	0.820	0.96	**0.053**
Age (reference: *65–69 years*)						
70–74 years	1.50	**< 0.001**	1.49	**< 0.001**	1.45	**< 0.001**
75–79 years	2.05	**< 0.001**	2.05	**< 0.001**	1.91	**< 0.001**
80–84 years	2.44	**< 0.001**	2.46	**< 0.001**	2.22	**< 0.001**
85 + years	2.52	**< 0.001**	2.62	**< 0.001**	2.39	**< 0.001**
Education (reference: *Primary*)						
Secondary	0.96	0.084	0.95	**0.038**	1.00	0.979
Tertiary	1.14	**< 0.001**	1.12	**< 0.001**	1.20	**< 0.001**
Marital status (reference: *Married*)						
Divorced	0.73	**< 0.001**	0.75	**< 0.001**	0.73	**< 0.001**
Widowed	0.86	**< 0.001**	0.88	**< 0.001**	0.86	**< 0.001**
Never	0.78	**< 0.001**	0.81	**< 0.001**	0.81	**< 0.001**
Employment (reference: *Retired*)						
Employed	0.74	**< 0.001**	0.75	**< 0.001**	0.79	**< 0.001**
Other	0.97	0.350	0.99	0.669	0.98	0.436
Income (reference: *< Q1*)						
Q1-Q2	1.15	**< 0.001**	1.14	**< 0.001**	1.15	**< 0.001**
Q2-Q3	1.25	**< 0.001**	1.23	**< 0.001**	1.27	**< 0.001**
Q3-Q4	1.33	**< 0.001**	1.30	**< 0.001**	1.37	**< 0.001**
Q4-Q5	1.26	**< 0.001**	1.24	**< 0.001**	1.33	**< 0.001**
Area of residence (reference: *Rural areas*)						
Cities	1.12	**< 0.001**	1.11	**< 0.001**	1.10	**< 0.001**
Towns and suburbs	1.04	**0.038**	1.04	0.065	1.04	0.084
BMI (reference: *Normal weight*)						
Underweight			0.88	**0.059**	0.86	**0.030**
Overweight			1.10	**< 0.001**	1.03	0.140
Obese			1.25	**< 0.001**	1.04	**0.081**
Smoking (reference: *Daily*)						
Former			1.46	**< 0.001**	1.44	**< 0.001**
Occasional			1.12	0.072	1.13	0.069
Never			1.27	**< 0.001**	1.31	**< 0.001**
Diet (reference: *Insufficient*)						
Moderate			1.14	**0.001**	1.17	**< 0.001**
Sufficient			1.35	**< 0.001**	1.40	**< 0.001**
Physical activity (reference: *Inactive*)						
Low			1.04	0.173	1.12	**< 0.001**
Moderate			1.07	**0.005**	1.22	**< 0.001**
High			1.08	**0.008**	1.31	**< 0.001**
Limitations (reference: *Not limited*)						
Limited					1.22	**< 0.001**
Severely limited					1.18	**< 0.001**
Asthma (reference: *Yes*)						
No					0.66	**< 0.001**
Bronchitis (reference: *Yes*)						
No					0.63	**< 0.001**
Blood pressure (reference: *Yes*)						
No					0.79	**< 0.001**
Coronary (reference: *Yes*)						
No					0.83	**< 0.001**
Heart attack (reference: *Yes*)						
No					0.90	**0.011**
Stroke (reference: *Yes*)						
No					0.99	0.826
Arthrosis (reference: *Yes*)						
No					0.82	**< 0.001**
Diabetes (reference: *Yes*)						
No					0.74	**< 0.001**
Bladder (reference: *Yes*)						
No					0.95	**0.057**
Kidney (reference: *Yes*)						
No					0.93	**0.052**
Depression (reference: *Yes*)						
No					0.94	**0.036**
**Random Effects**						
VPC*	0.258		0.258		0.256	
N _COUNTRY_	29		29		29	
Observations	77,941		77,941		77,941	
Marginal R^2^ / Conditional R^2^	0.036 / 0.267		0.043 / 0.270		0.067 / 0.308	

*Notes*: *Variance partitioning coefficient

In particular, there are strong significant differences between the 65–69 age group and the other older ages, which supports the idea that with age, elderly are more likely to be vaccinated against the influneza virus. Furthermore, the magnitude of the association with a higher level of education is lower compared to younger and older adults. When controlling only for socio-economic characteristics and health behavior factors, there was no gender effect concerning flu vaccination. On the other hand, adjusted for limitations and chronic conditions, the results associated with gender also hold for this age group, *i.e.* women were more likely to have had a flu vaccination in the past 12 months. In comparison with other age groups, marital status has a stronger effect among the elderly - those who are married have a higher probability of using vaccination as a prevention measure against influenza, whereas employed elderly are less likely to use the flu vaccine than those in other age groups. Income is also significantly associated with a higher likelihood of getting the flu vaccine, but the differences between income groups are more pronounced compared to the other three age groups. The results related to the association between flu vaccination utilization and BMI are no longer consistent in terms of significance of the coefficients. Considering the other variables related to personal health practices, the findings reveal that smoking, as well as an unbalanced diet or physical inactivity, reduces more the probability of using flu vaccination. Having difficulties in activities of daily living is also significantly associated with an increased probability of having a flu vaccination. Furthermore, a more pronounced association is found between different chronic diseases and the use of flu vaccination, highlighting the tendency to be more health-conscious with age. In addition, older people who suffer from depression are more likely to get the flu vaccine.

### Link between the use of flu vaccination and healthcare system

Irrespective of age group, the VPCs indicate that part of the between-country variance remains unexplained by the models including only the individual characteristics (Tables 1, 2, 3 and 4). Therefore, in a second step of the analysis, the effect of various country-level factors on flu vaccination uptake is separately examined. In this regard, all models control for the individual-level variables shown in Tables 1, 2, 3 and 4, but only odds ratios associated to health systems variables are provided (Table 5).

**Table 5:** The association between flu vaccination and country-level factors

Variables	Adolescents (*n* = 12,396)			Young Adults (*n* = 81,463)			Adults (*n* = 93,720)			Elderly (*n* = 77,941)		
	OR	*p*-value	VPC*	OR	*p*-value	VPC*	OR	*p*-value	VPC*	OR	*p*-value	VPC*
System (reference: *Beveridge*)												
Bismarck	1.09	0.789	0.156	0.63	0.100	0.143	0.67	0.137	0.138	0.44	**0.013**	**0.222**
Public healthcare expenditure	1.01	0.924	0.156	1.07	0.362	0.152	1.12	0.131	0.136	1.27	**0.011**	**0.220**
Out-of-pocket healthcare expenditure	1.01	0.737	0.154	1.01	0.771	0.154	0.98	0.219	0.140	0.96	**0.036**	**0.225**
Generalist** (reference: *< Q1*)												
Q1-Q2	1.20	0.674		1.31	0.470		1.69	0.142		1.95	0.169	
Q2-Q3	1.32	0.534		1.31	0.488		1.53	0.253		2.33	0.087	
> Q3	1.37	0.427	0.152	2.05	**0.035**	0.136	2.25	**0.013**	0.126	2.74	**0.023**	**0.223**
Specialist*** (reference: *< Q1*)												
Q1-Q2	1.61	0.266		1.81	0.105		1.57	0.197		1.19	0.711	
Q2-Q3	0.83	0.701		0.84	0.668		0.87	0.726		0.49	0.137	
> Q3	0.80	0.681	0.143	0.80	0.632	0.143	0.96	0.932	0.137	0.49	0.167	0.229
Primary care (reference: *No*)												
Yes	0.97	0.918	0.156	0.63	0.108	0.145	0.63	0.095	0.134	0.49	0.067	0.236
Portfolio of services (reference: *No*)												
Yes	0.75	0.357	0.154	0.92	0.755	0.156	1.06	0.834	0.146	1.18	0.655	0.254
Copayment (reference: *No*)												
Yes	1.38	0.292	0.152	1.23	0.460	0.154	0.99	0.981	0.147	0.64	0.227	0.252

*Notes*: Random intercept models adjusted for sex, age, education, marital status, employment, income, area of residence, BMI, smoking, diet, physical activity – for each age group; and self-perceived health – for adolescents; limitations, asthma, blood pressure, diabetes, depression – for young adults; limitations, asthma, bronchitis, blood pressure, coronary, diabetes, depression – for adults; limitations, asthma, bronchitis, blood pressure, coronary, heart attack, stroke, arthrosis, diabetes, bladder, kidney, depression – for elderly. *VPC – variance partitioning coefficient. **The number of generalist practitioners is grouped according to quartiles, for which the following values were obtained: Q1 = 74.7, Q2 = 88, Q3 = 99.95 per 100,000 inhabitants. ***The number of specialist practitioners is also grouped according to the quartile results as follows: Q1 = 209.6; Q2 = 248.5; Q3 = 317.1 per 100,000 inhabitants.

Several significant associations were found between the use of flu vaccination and country-level factors but only among the elderly. These results highlight that this segment of the population is indirectly affected by the characteristics of the healthcare system adopted in each country. In this respect, the results indicate that belonging to a country with a Bismarck-type healthcare system is associated with lower odds of getting a flu vaccination, in comparison with countries adopting the Beveridge healthcare system. Concerning the role of public funding, the results indicate that in countries where public health expenditure was higher (calculated as the share of GDP), the elderly are more likely to get a flu vaccination, while a higher level of out-of-pocket health expenditure discourages them to use this preventive service against influenza. Regarding the availability of doctors, the number of generalists (but only higher than Q3) is positively associated with the use of flu vaccination, for all age groups except adolescents. However, the number of specialists does not appear to have a significant association with the probability of getting a flu vaccination. Finally, irrespective to age group, no significant associations were found between the probability of using vaccination as a measure to prevent influenza and the other healthcare system characteristics. Of the significant macro-level determinants, the model with public healthcare expenditure (VPC reduced to 22%), followed by the one including the type of healthcare system (VPC reduced to 22.2%), explains most of the between-country variance in flu vaccine uptake among the elderly population. For both young and adult populations, the results indicate that most of the between-country variance in the likelihood of influenza vaccination is explained by the model including the number of general practitioners (VPC reduced to 13.6% for young adults; VPC reduced to 12.6% for adults).

## Discussion

This study examined socio-economic inequalities in using flu vaccination among the population in the 29 European countries using both individual- and country-level factors. Overall, the results confirm the existence of socio-economic disparities between individuals in different age groups (*i.e.* adolescents, young adults, older adults, and elderly), but also of significant variation between European countries, particularly for older people, in the uptake of influenza vaccination.

While the likelihood of getting a flu vaccination is positively correlated with age for both older adults and elderly, the probability of being vaccinated against flu is not significantly different between different age intervals corresponding to young adults age group. These findings are in line with other studies emphasizing that with age people become more health conscious and therefore may seek prevention measures more frequently than their younger counterparts [25]. With respect to elderly population, our findings are consistent with many previous studies highlighting that older people were more compliant regarding influenza vaccination than younger age groups [28–30, 76]. However, Lau *et al.* [77] found that people aged > 85 years were less likely to have been vaccinated than younger people.

On average, in the European countries included in this study, women have a higher propensity to use flu vaccination, except for the adolescents and elderly groups where there is no gender difference. There is other empirical evidence also suggesting that there are no gender differences in influenza vaccination in the population aged 50 years and over [22], which could be explained by the existence of other determinants with a stronger impact on using preventive health services. Nonetheless, Bödeker *et al.* [28] argue that vaccination coverage was higher among elderly females than elderly males. Therefore, further evidence is needed.

Regardless of age group, the results indicate that the use of influenza vaccination shows consistent and significant inequalities across all socio-economic indicators. In this regard, the use of influenza vaccine was significantly more common among respondents in higher socio-economic position. Therefore, on average across countries, controlling for individual characteristics and systemic differences at country level, individuals with higher levels of education and with higher incomes are more likely to use this kind of preventive care. These results are supported by previous studies which highlight that the use of flu vaccination is concentrated among higher income and better educated individuals in most EU countries [2, 78, 79]. A possible explanation is given by Cawley and Ruhm [32], who argue that lower socio-economic status, as proxied by educational attainment or income, is generally correlated with less healthier behaviors and therefore with poorer health. The same authors highlight that lower income also appears to be associated with unhealthy behaviors, independent of education. Moreover, people with little financial resources encounter difficulties buffering against the negative impacts of an adverse health, which can lead to poor health and in turn to a dangerous cycle of further impoverishment [80].

The same applies to other socio-economic variables, such as employment status and area of residence. In compliance with Voncina *et al.* [81] or Kim *et al.* [35], unemployment was found to be negatively associated with the use of vaccination immunization for both adults and elderly groups. In addition, Voncina *et al.* [81] suggest that unemployed people who have developed health-related problems or chronic health conditions have difficulty returning to work. Therefore, the possible inequity in the use of preventive healthcare among unemployed individuals may influence not only the health status but also their ability to return to work. This argument could be particularly relevant for countries with high unemployment rates. In this regard, in order to achieve a more equitable distribution of preventive healthcare services, the healthcare system of these countries should pay additional attention to the unemployed - for instance, by developing health prevention programs targeting this vulnerable population. In terms of the association between influenza vaccination and the area of residence, the results reveal that individuals from a rural area are the least preventive when it comes to using the flu vaccine. One possible explanation for these results is that rural residents have lower access to health information and health services [82, 83].

Marital status is another significant socio-economic determinant of influenza immunization, revealing that married people are more likely to be vaccinated compared to unmarried ones. This result is consistent with other studies arguing that being married is associated with more preventive and healthier behavior and therefore with better overall health [34, 84, 85]. In line with Lindström *et al.* [86], marital status should be viewed as a marker of risk, with the lowest health risks for those who are married/cohabiting. One possible factor why individuals would be more likely to receive preventive care, and which is well documented in previous literature [87], is that having a partner encourages individuals to keep medical appointments, checkups, *etc.* [84, 88]. The same argument is given by Kim *et al.* [34], who argue that family members, especially the spouse/partner, play an important role in disease prevention and health promotion. Contrary to the general belief that the health benefits of marriage accrue mainly to men, Miller and Pylypchuk [85] found that marriage increases preventive care utilization for both sexes. Nevertheless, as Bookwala [89] underlines, uncaring and unhelpful spousal behaviors can outweigh positive spousal behaviors and contribute to health neglect and therefore poorer health.

Except for adolescents’ group, the analysis provided similar results concerning the association between personal health practices and the probability of getting a flu vaccination. Findings indicate that people who smoke, have problems with obesity, do not have a balanced diet, or do not engage in any physical activity are less likely to use flu vaccination. Previous studies support the notion that bad health habits were associated with vaccination refusal [21, 23, 31, 90, 91]. Additionally, health motivation and health consciousness are also shown to influence preventive healthcare behaviors [30]. And most of the time health consciousness comes from the constraint of poor health or the presence of certain diseases, an argument that is sustained by the results on the association of (self-perceived) health status with flu vaccination use. Irrespective of age group, it is observed that poor health status – driven by different degrees of difficulty in activities of daily living and different chronic diseases – encouraged the use of vaccination, while good health was the most common reason for not getting the flu vaccine. However, as found in many other studies conducted on the population from different European countries [28, 29, 92–94], this association is more consistent and significant with age, which could be explained by the fact that, as people get older, their health declines and, as a result, they become more preventive. Another point of view is given by Carrieri and Wübker [2] who argue that health-related factors that have a positive impact on the uptake of preventive healthcare may be concentrated among lower income groups and may capture some of the correlation between income and preventive measures. These results suggest that more attention needs to be paid to low-income elderly groups in the design of effective prevention programs.

This argument is sustained to some extent to the country-level systemic differences in terms of flu vaccination use. However, in compliance with Kino *et al.* [33], the random statistics of the models indicate the substantial role of individual-level rather than country-level factors in explaining variations in flu vaccination uptake. Consistent and significant associations between flu vaccine use and country-level factors – such as the type of healthcare system adopted in each of the 29 European countries, the public health funding, the out-of-pocket health expenditure, and the availability of doctors – were found especially among the elderly population. For instance, countries with a Bismarck-type healthcare system are associated with lower rates of flu vaccination, in comparison with countries adopting the Beveridge healthcare system. According to the European Centre for Disease Prevention and Control [12], all EU countries recommended the vaccine for targeted population, including those aged 65 years and over. In this respect, some countries (as Italy, Germany, Greece, Hungary, Iceland, and the Netherlands) have even lowered the age limit in elderly adults. However, this recommendation has not been funded or has been partially funded in some of the countries, including Austria, Bulgaria, Estonia, Latvia, Poland, and Slovenia. As a consequence, in all of these countries where co-payment is required, influenza vaccination rates were among the lowest in the EU. With the exception of Latvia, the healthcare system of all the other countries is based on the Bismarck model, which does not provide universal coverage. Therefore, the reasoning behind this finding is also linked, to some extent, to the type of financing approached by the two healthcare systems and their beneficiary population: the Bismarck health system - compulsory funded by employers and employees, administered by pre-existing “sickness funds”, and not aiming universal coverage - *vs.* the Beveridge health system - funded from general government revenues and providing coverage for entire population. We can conclude that these arguments also support the impact of the other factors on the use of influenza vaccine among the elderly population.

In terms of public investment in health, the share of public health expenditure in GDP level appears to have a direct impact on the use of influenza vaccination among elderly population. In countries with the lowest level of spending on preventive healthcare, vaccination rates, especially among the elderly population, are also among the lowest. Most of these countries (Bulgaria, Poland, Estonia, Slovenia, Czech Republic, and Romania) follow the Bismarck health system. Another significant link appears to be between the use of preventive care and the share of out-of-pocket expenditure in total health expenditure, which is an indicator measuring the direct cost of care for individuals. Thus, covering all or part of the costs out of their own pocket discourages people, especially the elderly, from using such preventive healthcare services. Furthermore, in countries with high numbers of generalists, higher prevention rates are also recorded, a result which seems to be valid for all age groups, except for adolescents. A possible explanation is given by Jusot *et al.* [22] who argue that in these countries general practitioners may have more time to spend with patients, which, in turn, could provide more prevention opportunities. The same authors also highlight the important role of generalists in ensuring appropriate primary prevention, such as flu vaccination after a certain age. However, their findings do not support any significant association between health system characteristics and the propensity to use influenza vaccination across the countries studied. A possible explanation could be related to the sample the authors analyze, as it includes only 14 European countries.

### Strengths and limitations

The study faces some limitations. First, the use of cross-sectional data cannot determine whether individual-level predictors preceded influenza vaccination use. Therefore, we cannot discuss causality between these factors and vaccination among the EU population. Second, the survey did not include information that differentiates whether the respondents’ decision to vaccinate is a personal one, or one based on a recommendation from a health professional, or one that comes from the constraint of having certain medical conditions, which could have an impact on the findings. From this point of view, we believe that the nature of preventive services in general should be independent of an existing medical condition. Third, due to the lack of such information in the survey, the analysis does not explicitly capture the impact of certain factors related to individuals’ perceptions of influenza vaccine use. Finally, there is always the possibility of self-report bias in surveys, particularly in relation to certain individual characteristics and the use of preventive services.

Despite these limitations, we consider that the current study has direct implications for health policy. In this respect, the results reveal that socio-economic inequalities in the use of influenza vaccination are a Europe-wide problem, rather than a country-specific phenomenon. Limited vaccine uptake by people of lower socio-economic status, especially among the elderly population, means less primary preventive care that may lead to serious communicable diseases or even exacerbation of certain underlying comorbid conditions, such as cardiovascular disease, chronic respiratory diseases, diabetes, obesity, neurologic conditions, and bacterial co-infections, which are particularly susceptible to influenza infections [95, 96]. Therefore, all these consequences are further associated with a substantial clinical, humanistic, and economic burden [97]. In addition, the results also support the influence of some country-level factors on influenza vaccination uptake, again with an emphasis on elderly population. Despite the efforts of all EU countries to inform and encourage the population through specific recommendations to be vaccinated against influenza, the vaccination coverage rate remains below the 75% vaccination coverage rate target set by the EU for recommended target groups such as the elderly and those with underlying health conditions [12].

Besides the individual and country-level characteristics significantly associated with the use of flu vaccine, the European Commission also draws attention to declining public confidence in vaccination and to increasing misinformation and disinformation about vaccination. In this regard, the studies examining barriers and attitudes towards influenza vaccine uptake conclude that there is still significant hesitancy about influenza vaccine due to perceived low risk of illness combined with concerns about safety and efficacy [98, 99]. For instance, the most widely reported barriers to vaccination against influenza for the adult general population are the lack of trust in healthcare services, a perceived lack of knowledge of the influenza vaccine and the fear of vaccine-associated side effects. However, according to Welch *et al.* [99], there is a potential correlation between the fear of vaccine-associated side effects and poor knowledge of vaccine safety, which may lead to a delay or refusal of vaccination. Low vaccination coverage rates could also be explained by low flu risk perception, which influences the decision to get the flu vaccine. Among young adults, the most common reasons given for not getting the flu vaccine were the perception that “I am unlikely to get very sick with the flu” and that “I never get the flu” [55], or “I don’t need it because I am healthy” [57]. These findings underline that government actions to garner trust are essential to the success of flu vaccination programmes for people of all ages.

## Conclusions

This study shows that socio-economic inequalities in influenza vaccine uptake are present within each age group and are maintained even after controlling other behavioral and health status factors. If among adolescents, income is one of the most important factors explaining vaccine uptake, for the other age groups, in addition to income, education is another strong proxy of socio-economic status associated with the use of flu vaccination. Furthermore, these disparities within each population group are also explained by area of residence and occupational status. Therefore, our results suggest that recommendations to use the influenza vaccine should be extended to the younger population, given that within each age group the likelihood of using the influenza vaccine is reduced by disadvantaged socio-economic conditions. At the same time, especially for the elderly population, the differences between individuals in vaccine utilization are also supported by macro-contextual factors, such as the type of healthcare system adopted in each country, public funding, personal health expenditure burden, or the availability of generalist practitioners. From this perspective, the vulnerability of this category of the population and its higher dependence on the public health system is highlighted. Our findings reveal that vaccination against seasonal influenza remains a critical public health intervention and bring attention to the relevance of conceiving and implementing context-specific strategies to ensure equitable access to vaccines for all EU citizens.

### Electronic supplementary material

Below is the link to the electronic supplementary material.

Additional file 1. Appendix: Table A1a. Sample structure by use of flu vaccination used and country. Table A1b. Sample structure by use of flu vaccination used and age groups. Table A2. Sample structure by individual characteristics and age groups. Table A3. Sample structure by country-level characteristics and flu vaccination use.

## Data Availability

This paper is based on data from Eurostat, European Health Interview Survey (EHIS) 2019. We obtained these data by applying a research project proposal at institutional level, according to the criteria required by Eurostat, as mentioned on the official webpage: https://ec.europa.eu/eurostat/web/microdata/european-health-interview-survey. The EHIS 2019 data are official, public, and institutionally available from Eurostat. The responsibility for all conclusions drawn from the data lies entirely with the authors.

## Abbreviations

BMI: Body Mass Index
ECDC: European Centre for Disease Prevention and Control
EHIS: European Health Interview Survey
IPAQ-SF: International Physical Activity Questionnaire - Short Form
MET: Metabolic Equivalent
