# Supplementary material for: Socio-economic inequalities in the use of flu vaccination in Europe: a multilevel approach
Source: Health Econ Rev. 2024 Jul 31;14:61. doi: 10.1186/s13561-024-00535-1 (PMC11292999; doi:10.1186/s13561-024-00535-1)
Supplement: Supplementary file 1 — Additional file 1. Appendix: Table A1a. Sample structure by use of flu vaccination used and country. Table A1b. Sample structure by use of flu vaccination used and age groups. Table A2. Sample structure by individual characteristics and age groups. Table A3. Sample structure by country-level characteristics and flu vaccination use. [file 13561_2024_535_MOESM1_ESM.docx]

**Appendix**

**Table A1a**. Sample structure by use of flu vaccination and country

| **Country** | **Flu vaccination** | **Country** | **Flu vaccination** |
| --- | --- | --- | --- |
| AT | N = 15423 | IS | N = 2986 |
| Yes | 1323 (8.6%) | Yes | 1211 (40.6%) |
| No | 14100 (91.4%) | No | 1775 (59.4%) |
| BE | N = 6488 | IT | N = 42348 |
| Yes | 1614 (24.9%) | Yes | 8455 (20.0%) |
| No | 4874 (75.1%) | No | 33893 (80.0%) |
| BG | N = 6830 | LT | N = 4216 |
| Yes | 208 (3.0%) | Yes | 342 (8.1%) |
| No | 6622 (97.0%) | No | 3874 (91.9%) |
| CY | N = 5722 | LU | N = 2560 |
| Yes | 544 (9.5%) | Yes | 401 (15.7%) |
| No | 5178 (90.5%) | No | 2159 (84.3%) |
| CZ | N = 6892 | LV | N = 5700 |
| Yes | 729 (9.6%) | Yes | 506 (8.9%) |
| No | 6892 (90.6%) | No | 5194 (91.1%) |
| DE | N = 19684 | MT | N = 3941 |
| Yes | 5757 (29.2%) | Yes | 977 (24.8%) |
| No | 13927 (70.8%) | No | 2964 (75.2%) |
| DK | N = 4805 | NL | N = 7234 |
| Yes | 950 (19.8%) | Yes | 1898 (26.2%) |
| No | 3855 (80.2%) | No | 5336 (73.8%) |
| EE | N = 4764 | NO | N = 7254 |
| Yes | 635 (13.3%) | Yes | 1315 (18.1%) |
| No | 4129 (86.7%) | No | 5939 (81.9%) |
| EL | N = 7639 | PL | N = 12149 |
| Yes | 2661 (34.8%) | Yes | 818 (6.7%) |
| No | 4978 (65.2%) | No | 11331 (93.3%) |
| ES | N = 19869 | PT | N = 13547 |
| Yes | 4801 (24.2%) | Yes | 3067 (22.6%) |
| No | 15068 (75.8%) | No | 10480 (77.4%) |
| FI | N = 3883 | RO | N = 15127 |
| Yes | 1533 (39.5%) | Yes | 2085 (13.8%) |
| No | 2350 (60.5%) | No | 13042 (86.2%) |
| FR | N = 14143 | SE | N = 7526 |
| Yes | 2865 (20.3%) | Yes | 1123 (14.9%) |
| No | 11278 (79.7%) | No | 6403 (85.1%) |
| HR | N = 3057 | SI | N = 8533 |
| Yes | 561 (18.3%) | Yes | 566 (6.7%) |
| No | 2496 (81.7%) | No | 7967 (93.3%) |
| HU | N = 5095 | SK | N = 5393 |
| Yes | 655 (12.9%) | Yes | 388 (7.2%) |
| No | 4440 (87.1%) | No | 5005 (92.8%) |
| IE | N = 1983 |  |  |
| Yes | 692 (34.9%) |  |  |
| No | 1291 (65.1%) |  |  |

**Table A1b**. Sample structure by use of flu vaccination and age groups

| Flu vaccination | **Adolescents**  **(15-19 years)**  N = 12396 | **Adults Young**  **(20-44 years)**  N = 81463 | **Adults**  **(45-19 years)**  N = 93720 | **Elderly**  **(65+ years)**  N = 77941 |
| --- | --- | --- | --- | --- |
| Yes | 896 (5.95%) | 6056 (7.44%) | 12357 (13.19 %) | 29529 (37.89%) |
| No | 14249 (94.05%) | 75407 (92.56%) | 81363 (86.81%) | 48412 (62.11%) |
| *p-value** | *<0.001* | *<0.001* | *<0.001* | *<0.001* |
| *Notes: ** Chi-squared test was used for comparisons. | | | | |

**Table A2.** Sample structure by individual characteristics and age groups

| **Variables***** | **Adolescents**  **(15-19 years)**  N = 12396 | **Young Adults**  **(20-44 years)**  N = 81463 | **Adults**  **(45-64 years)**  N = 93720 | **Elderly**  **(65+ years)**  N = 77941 |
| --- | --- | --- | --- | --- |
| Age |  |  |  |  |
| 20-24 |  | 12469 (15%) |  |  |
| 25-29 |  | 13785 (17%) |  |  |
| 30-34 |  | 16033 (20%) |  |  |
| 35-39 |  | 18335 (23%) |  |  |
| 40-44 |  | 20841 (26%) |  |  |
| 45-49 |  |  | 21978 (23%) |  |
| 50-54 |  |  | 23551 (25%) |  |
| 55-59 |  |  | 23801 (25%) |  |
| 60-64 |  |  | 24390 (26%) |  |
| 65-69 |  |  |  | 23469 (30%) |
| 70-74 |  |  |  | 20189 (26%) |
| 75-79 |  |  |  | 12572 (16%) |
| 80-84 |  |  |  | 15869 (20%) |
| 85+ |  |  |  | 5842 (7.5%) |
| *p-value** |  | *<0.001* | *<0.001* | *<0.001* |
| Sex |  |  |  |  |
| Female | 6193 (50%) | 42655 (52%) | 49937 (53%) | 44204 (57%) |
| Male | 6203 (50%) | 38808 (48%) | 43783 (47%) | 33737 (43%) |
| *p-value** | *<0.001* | *<0.001* | *<0.001* | *<0.001* |
| Marital status |  |  |  |  |
| Divorced |  | 3793 (4.6%) | 12428 (13%) | 5859 (7.5%) |
| Married |  | 35212 (43.2%) | 62554 (67%) | 43877 (56%) |
| Never |  | 42458 (52.2%) | 14482 (15%) | 4753 (6.1%) |
| Widowed |  | - | 4256 (4.5%) | 23452 (30%) |
| *p-value** |  | *<0.001* | *<0.001* | *<0.001* |
| Area of residence |  |  |  |  |
| Cities | 4190 (34%) | 31738 (39%) | 31760 (34%) | 26826 (34%) |
| Rural areas | 3967 (32%) | 22904 (28%) | 30234 (32%) | 25458 (33%) |
| Towns and suburbs | 4239 (34%) | 26821 (33%) | 31726 (34%) | 25657 (33%) |
| *p-value** | *<0.001* | *<0.001* | *<0.001* | *<0.001* |
| Education |  |  |  |  |
| Primary | 1420 (11%) | 2817 (3.5%) | 8137 (8.7%) | 22300 (29%) |
| Secondary | 10976 (89%) | 44931 (55%) | 57230 (61%) | 40310 (52%) |
| Tertiary | - | 33715 (41.5%) | 28353 (30%) | 15331 (20%) |
| *p-value** | *<0.001* | *<0.001* | *<0.001* | *<0.001* |
| Income |  |  |  |  |
| <Q1 | 3402 (27%) | 13960 (17%) | 15030 (16%) | 15092 (19%) |
| Q1-Q2 | 2426 (20%) | 14038 (17%) | 15641 (17%) | 20173 (26%) |
| Q2-Q3 | 2400 (19%) | 16739 (21%) | 18646 (20%) | 16817 (22%) |
| Q3-Q4 | 2343 (19%) | 18492 (23%) | 21420 (23%) | 13863 (18%) |
| Q4-Q5 | 1825 (15%) | 18234 (22%) | 22983 (25%) | 11996 (15%) |
| *p-value** | *<0.001* | *<0.001* | *<0.001* | *<0.001* |
| Employment |  |  |  |  |
| Employed |  | 61048 (75%) | 64120 (68%) | 3578 (4.6%) |
| Retired |  |  | 11878 (13%) | 66969 (86%) |
| Unemployed |  | 6612 (8%) | 6012 (6.4%) | - |
| Other** |  | 13803 (17%) | 11710 (12%) | 7394 (9.5%) |
| *p-value** |  | *<0.001* | *<0.001* | *<0.001* |
| BMI |  |  |  |  |
| Underweight | 1469 (12%) | 2704 (3.3%) | 1254 (1.3%) | 1126 (1.4%) |
| Normal weight | 8954 (72%) | 45010 (55%) | 36061 (38%) | 27019 (35%) |
| Overweight | 1973 (16%) | 24416 (30%) | 37789 (40%) | 33989 (44%) |
| Obese | - | 9333 (11%) | 18616 (20%) | 15807 (20%) |
| *p-value** | *<0.001* | *<0.001* | *<0.001* | *<0.001* |
| Smoking |  |  |  |  |
| Daily | 989 (8.0%) | 17612 (22%) | 20307 (22%) | 7132 (9.2%) |
| Occasional | 799 (6.4%) | 5859 (7%) | 4079 (4.4%) | 1633 (2.1%) |
| Former | 362 (2.9%) | 11355 (14%) | 20700 (22%) | 21202 (27%) |
| Never | 10246 (83%) | 46637 (57%) | 48634 (52%) | 47974 (62%) |
| *p-value** | *<0.001* | *<0.001* | *<0.001* | *<0.001* |
| Diet |  |  |  |  |
| Insufficient | 1320 (11%) | 6908 (8.5%) | 6691 (7.1%) | 4984 (6.4%) |
| Moderate | 4039 (33%) | 23303 (28.5%) | 25017 (27%) | 19179 (25%) |
| Sufficient | 7037 (57%) | 51252 (63%) | 62012 (66%) | 53778 (69%) |
| *p-value** | *<0.001* | *<0.001* | *<0.001* | *<0.001* |
| Physical activity |  |  |  |  |
| Inactive | 658 (5.3%) | 8080 (10%) | 11802 (13%) | 16144 (21%) |
| Low | 1149 (9.3%) | 10628 (13%) | 13968 (15%) | 14230 (18%) |
| Moderate | 4663 (38%) | 34951 (43%) | 41213 (44%) | 31703 (41%) |
| High | 5926 (48%) | 27804 (34%) | 26737 (29%) | 15864 (20%) |
| *p-value** | *<0.001* | *<0.001* | *<0.001* | *<0.001* |
| Self-perceived health |  |  |  |  |
| Bad | - |  |  |  |
| Fair | 875 (7.1%) |  |  |  |
| Good | 4483 (36%) |  |  |  |
| Very good | 7038 (57%) |  |  |  |
| *p-value** | *<0.001* |  |  |  |
| Limitations |  |  |  |  |
| Not limited |  | 71765 (88%) | 68342 (73%) | 40368 (52%) |
| Limited |  | 7928 (9.7%) | 19561 (21%) | 26188 (34%) |
| Severely limited |  | 1770 (2.3%) | 5817 (6%) | 11385 (15%) |
| *p-value** |  | *<0.001* | *<0.001* | *<0.001* |
| Asthma |  |  |  |  |
| Yes |  | 3747 (4.6%) | 4740 (5.1%) | 4816 (6.2%) |
| No |  | 77716 (95.4%) | 88980 (94.9%) | 73125 (93.8%) |
| *p-value** |  | *<0.001* | *<0.001* | *<0.001* |
| Bronchitis |  |  |  |  |
| Yes |  |  | 3447 (3.7%) | 6098 (7.8%) |
| No |  |  | 90273 (96.3%) | 71843 (92.2%) |
| *p-value** |  |  | *<0.001* | *<0.001* |
| Coronary |  |  |  |  |
| Yes |  |  | 2330 (2.5%) | 7226 (9.3%) |
| No |  |  | 91390 (97.5%) | 70715 (90.7%) |
| *p-value** |  |  | *<0.001* | *<0.001* |
| Blood_pressure |  |  |  |  |
| Yes |  | 3604 (4.4%) | 23436 (25%) | 40238 (52%) |
| No |  | 77859 (95.6%) | 70284 (75%) | 37703 (48%) |
| *p-value** |  | *<0.001* | *<0.001* | *<0.001* |
| Diabetes |  |  |  |  |
| Yes |  | 954 (1.2%) | 6437 (6.9%) | 13802 (18%) |
| No |  | 80509 (98.8%) | 87283 (93.1%) | 64139 (82) |
| *p-value** |  | *<0.001* | *<0.001* | *<0.001* |
| Depression |  |  |  |  |
| Yes |  | 4720 (5.2%) | 6966 (7.4%) | 6640 (8.5%) |
| No |  | 77193 (94.8%) | 86754 (92.6%) | 71301 (91.5%) |
| *p-value** |  | *<0.001* | *<0.001* | *<0.001* |
| Heart_attack |  |  |  |  |
| Yes |  |  |  | 3574 (4.6%) |
| No |  |  |  | 74367 (95.4%) |
| *p-value** |  |  |  | *<0.001* |
| Stroke |  |  |  |  |
| Yes |  |  |  | 3008 (3.9%) |
| No |  |  |  | 74933 (96.1%) |
| *p-value** |  |  |  | *<0.001* |
| Arthrosis |  |  |  |  |
| Yes |  |  |  | 27877 (36%) |
| No |  |  |  | 50064 (64%) |
| *p-value** |  |  |  | *<0.001* |
| Bladder |  |  |  |  |
| Yes |  |  |  | 10359 (13%) |
| No |  |  |  | 67582 (87%) |
| *p-value** |  |  |  | *<0.001* |
| Kidney |  |  |  |  |
| Yes |  |  |  | 4906 (6.3%) |
| No |  |  |  | 73035 (93.7%) |
| *p-value** |  |  |  | *<0.001* |
| *Notes: ** Chi-squared test was used for comparisons. *** Other* category includes: unable to work due to longstanding health problems; student, pupil; fulfilling domestic tasks; compulsory military or civilian service. ******* We have presented the descriptive statistics only for the variables included in the models for each age group. | | | | |

**Table A3.** Sample structure by country-level characteristics and flu vaccination use

| **Country-level** | **N (%)** | **Flu vaccination** | | | |
| --- | --- | --- | --- | --- | --- |
|  |  | **Uptake (%)** | **No uptake (%)** | | |
| Healthcare system |  |  | |  | |
| Beveridge | 119564 (45.03) | 25174 (21.05) | | 94390 (78.95) | |
| Bismarck | 145956 (54.97) | 23506 (16.10) | | 122450 (83.90) | |
| *p-value** | *<0.001* | *<0.001* | | *<0.001* | |
| Generalist practitioners |  |  | |  | |
| <Q1 | 69472 (26.16) | 8865 (12.76) | | 60607 (87.24) | |
| Q1-Q2 | 74042 (27.89) | 13663 (18.45) | | 60379 (81.55) | |
| Q2-Q3 | 55089 (20.75) | 12818 (23.27) | | 42271 (76.73) | |
| >Q3 | 66917 (25.20) | 13334 (19.93) | | 53583 (80.07) | |
| *p-value** | *<0.001* | *<0.001* | | *<0.001* | |
| Specialist practitioners |  |  | |  | |
| <Q1 | 54755 (20.62) | 9626 (17.58) | | 45129 (82.42) | |
| Q1-Q2 | 69119 (26.03) | 13611 (19.69) | | 55508 (80.31) | |
| Q2-Q3 | 89934 (33.87) | 15202 (16.90) | | 74732 (83.10) | |
| >Q3 | 51712 (19.48) | 10241 (19.80) | | 41471 (80.20) | |
| *p-value** | *<0.001* | *<0.001* | | *<0.001* | |
| Primary care |  |  | |  | |
| Yes | 193201 (72.76) | 33902 (17.55) | | 159299 (82.45) | |
| No | 72319 (27.24) | 14778 (20.43) | | 57541 (79.57) | |
| *p-value** | *<0.001* | *<0.001* | | *<0.001* | |
| Portfolio of services |  |  | |  | |
| Yes | 82958 (31.24) | 14891 (17.95) | | 68067 (82.05) | |
| No | 182562 (68.76) | 33789 (18.51) | | 148773 (81.49) | |
| *p-value** | *<0.001* | *<0.001* | | *<0.001* | |
| Copayment |  |  | |  | |
| Yes | 129423 (48.74) | 24150 (18.66) | | 105273 (81.34) | |
| No | 136097 (51.26) | 24530 (18.02) | | 111567 (81.98) | |
| *p-value** | *<0.001* | *<0.001* | | *<0.001* | |
|  | **Mean** | **S.D.** | | **Min** | **Max** |
| Public healthcare expenditure | 6.62 | 1.76 | | 3.86 | 9.83 |
| Out-of-pocket healthcare expenditure | 20.51 | 7.53 | | 9.46 | 37.8 |
| *Notes: ** Chi-squared test was used for comparisons. | | | | | |
